# The application and accuracy of feature matching on automated cephalometric superimposition

**DOI:** 10.1186/s12880-020-00432-z

**Published:** 2020-03-19

**Authors:** Yiran Jiang, Guangying Song, Xiaonan Yu, Yuanbo Dou, Qingfeng Li, Siqi Liu, Bing Han, Tianmin Xu

**Affiliations:** 1 Department of Orthodontics, Peking University School and Hospital of Stomatology, 22 Zhongguancun South Street, Haidian District, Beijing, 100081 China; 2 State Key Laboratory of Virtual Reality Technology and Systems, School of Computer Science and Engineering, Beihang University, Beijing, China; 3 Beijing Advanced Innovation Center for Big Data and Brain Computing (BDBC), Beihang University, Beijing, China; 4 Hangzhou Innovation Research Institute, Beihang University, Beijing, China; 5 First Clinical Division, Department of Orthodontics, Peking University School and Hospital of Stomatology, Beijing, China

**Keywords:** Digital imaging/radiology, Orthodontic(s), Cephalometric superimposition, Feature matching, Accuracy

## Abstract

**Background:**

The aim of this study was to establish a computer-aided automated method for cephalometric superimposition and to evaluate the accuracy of this method based on free-hand tracing.

**Methods:**

Twenty-eight pairs of pre-treatment (T_1_) and post-treatment (T_2_) cephalograms were selected. Structural superimpositions of the anterior cranial base, maxilla and mandible were independently completed by three operators performing traditional hand tracing methods and by computerized automation using the feature matching algorithm. To quantitatively evaluate the differences between the two methods, the hand superimposed patterns were digitized. After automated and hand superimposition of T_2_ cephalograms to T_1_ cephalometric templates, landmark distances between paired automated and hand T_2_ cephalometric landmarks were measured. Differences in hand superimposition among the operators were also calculated.

**Results:**

The T_2_ landmark differences in hand tracing between the operators ranged from 0.61 mm to 1.65 mm for the three types of superimposition. There were no significant differences in accuracy between hand and automated superimposition (*p* > 0.05).

**Conclusions:**

Computer-aided cephalometric superimposition provides comparably accurate results to those of traditional hand tracing and will provide a powerful tool for academic research.

## Background

Since the introduction of cephalometry in 1931 by Broadbent [1], it has become an important tool in clinical diagnosis, treatment planning, evaluation of treatment changes and growth study. The traditional method of cephalometric analysis is hand tracing the craniofacial soft and hard anatomic structural contours on cephalograms on acetate paper. This process is subjective, and the accuracy varies with personal experience, knowledge, understanding of craniofacial anatomy and tracking preference [2–6]. Additionally, this process is time-consuming, and the accuracy is also inevitably influenced by the human fatigue level [7, 8]. In particular, for the purpose of research, a certain number of cephalograms need to be traced and measured within a certain time constraint, and the intra−/inter-reproducibility is impacted.

With the development of digital technology, the traditional hand tracing cephalogram is being replaced by digital cephalometric analysis. In previous studies, the latter method, using commercial software, has been proven to be accurate, reliable and time-saving [8–12]. However, the only exception is the structural superimposition for treatment evaluation and growth study, although much effort has been made in this field [3, 12–14]. The reason for this exception lies in the fact that landmark identification is easily accomplished using commercial software, while structural superimposition focuses on the tracing of structural details, which is independent of landmarks and is still not feasible using current commercial software.

Baumrind et al. believed that hand superimpositions resulted in better quality than any computer-aided superimpositions, because biological craniofacial growth is difficult to be interpreted by any mathematical equations [2]. However, due to the absolute consistency, fully automated cephalometric analysis has always been a popular challenge in computer science. One of these methods is the knowledge-based line extraction technique [15], which duplicates the strategy of orthodontists by extracting important anatomic edges and locating landmarks according to geometric definitions. However, the irregular details of bone, such as the inter-trabeculae, incisor nerve canal and inferior alveolar canal, make computer automated tracing difficult and questionable. Other studies have attempted to locate landmarks directly [16, 17], and the techniques have evolved from template matching [16] to, more recently, neural network models [17]. In terms of structural superimposition on stable regions instead of reference planes, which has been recognised as the most accurate method [18–22], why not use the same strategy?

Feature matching is a computer algorithm [23, 24] whose mission is to detect and match keypoints of the same or similar regions in multiple images taken at different viewpoints, under different illuminations, or at different magnifications. In comparison with the traditional manual process of superimposing the stable structures of two serial cephalograms, this method bears many similarities. One of the algorithms, Oriented FAST and Rotated BRIEF(ORB) [23], was shown to be time-saving for the matching process, rotational-invariant and noise-immune. However, two aspects of this method should be improved for clinical applications: (1) the area for detecting and matching keypoints should be limited to the stable regions on the cephalograms; and (2) to achieve accurate matching results, the matches should be not only abundant but also of high quality.

Currently, studies describing methods for automated cephalometric structural superimposition are scarce. Therefore, the present study aimed to (1) establish a computer-aided automated method of structural superimposition on the anterior cranial base, maxilla and mandible and (2) evaluate its accuracy based on free-hand tracing and superimposition.

## Methods

The institutional review board for the protection of human subjects reviewed and approved the research protocol (IRB-201626016).

**Fig. 1:**
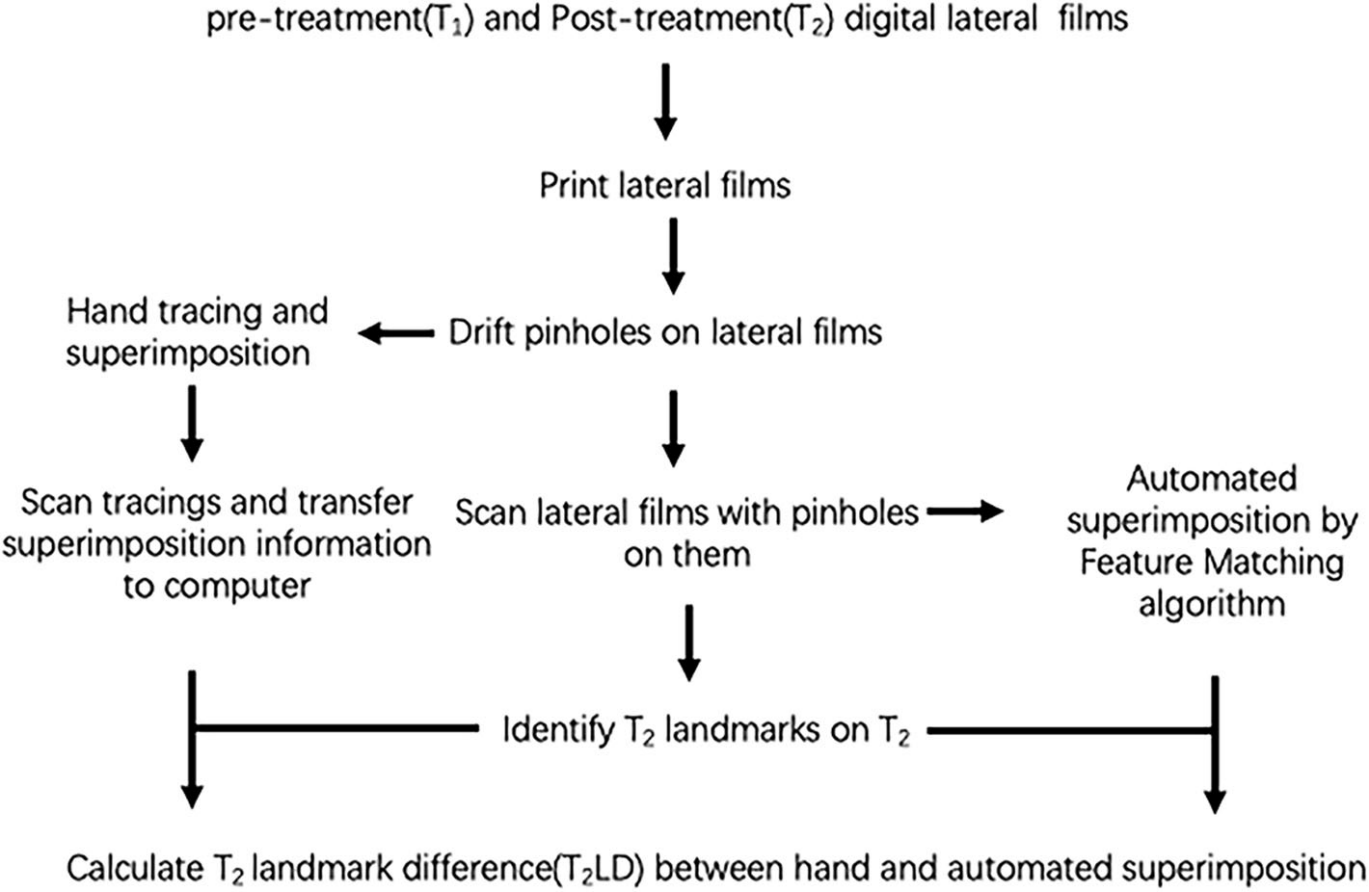
Schematic flowchart of the experimental design

A total of 28 pairs of pre- (T_1_) and post-treatment (T_2_) cephalograms were selected. They were taken by the same X-ray machine. The subjects consisted of 21 females and 7 males. The age for T_1_ ranged from 12 to 27 years, with a mean age of 15.32 years; the T_2_ ages ranged from 14 to 29 years, with a mean age of 18.03 years. The experimental design is shown in Fig. 1. Calibration rulers were used to control distortions and resolution errors during the printing and scanning process.

### Landmark identifications

One operator identified landmarks on the T_1_ and T_2_ digital lateral films using customised software produced by the State Key Laboratory of Virtual Reality Technology and Systems. The landmarks included the upper reference point (URP), lower reference point (LRP), sella (S), nasion (N), pterygoid point (Pt), posterior nasal spine (PNS), anterior nasal spine (ANS), subspinale (A), supramental (B), pogonion (Pg), menton (Me), gonion (Go), condylion (Co), upper incisor edge (UIE), upper incisor root apex (UIA), upper first molar mesial buccal cusp (UM), upper first molar mesial root apex (UMA), lower incisor edge (LIE), lower incisor apex (LIA), lower first molar mesial buccal cusp (LM), and lower first molar mesial apex (LMA).

### Superimposition methodology

The structural superimposition method developed by Johnston [25] for the anterior cranial base, maxilla and mandible was independently conducted by each operator.

### Hand tracing superimposition

Three senior orthodontic residences, who finished the superimposition course in our department and attended the hands-on lecture given by Johnston in person, were selected as operators in this study. They independently performed hand tracings of T_1_ and T_2_ side by side on acetate paper. Information from the hand superimposition was recorded using a similar method as that developed by the University of California, San Francisco [11]. A series of ten pinholes were drilled into T_1_ films in the non-information-bearing area surrounding the anatomic region of interest. Four corner pinholes on the T_1_ films, called positioning reference pinholes, were used to register the scanned hand tracings onto corresponding digital films. The other six pinholes on the T_1_ films, called superimposing reference pinholes, were used in pairs to register the between-film relationships onto T_2_ tracings for the three types of hand superimposition methods and to convert the between-film relationship of the hand superimpositions into a digital record by scanning. For the T_2_ films, only the four corner pinholes were drilled. The lateral films with pinholes and the corresponding hand tracings were scanned (HP Color LaserJet 2840, Hewlett-Packard Company, Palo Alto, CA, USA) in original size and at 600 dpi. The operators identified the scanned pinholes that carried the information on superimposition and image size from the T_1_ and T_2_ tracings.

### Automated superimposition

**Fig. 2:**
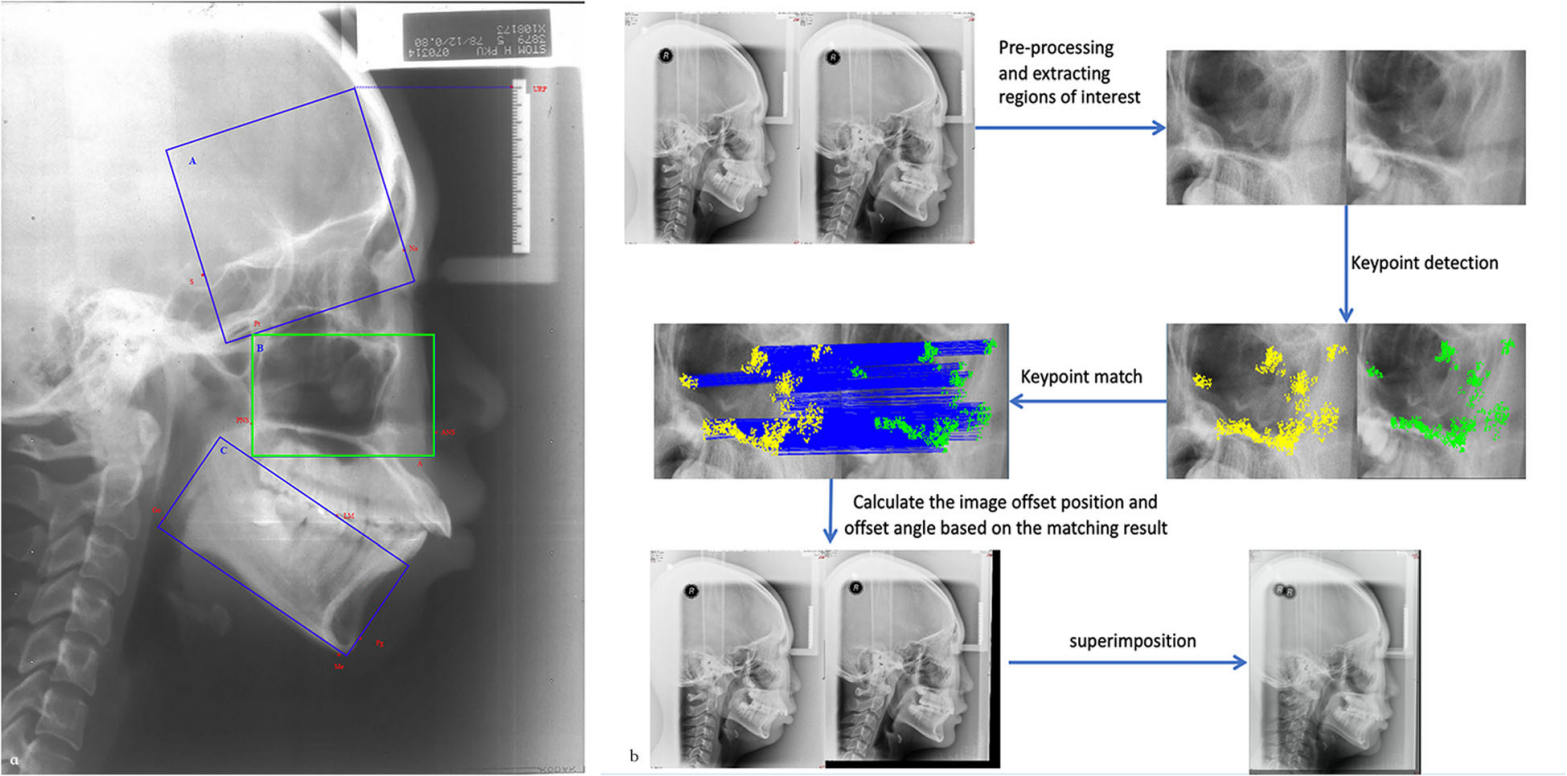
**a** The rectangular areas mainly consisting of stable structures were used to detect keypoints enclosed by landmarks on the anterior cranial base, maxilla and mandible. A. Area on the Anterior cranial base: URP-S-Pt-Na. B. Area on the maxilla: Pt-PNS-ANS-A. C. Area on the mandible: LM-Pg-Me-Go. **b** The flowchart of feature matching process and automated superimposition on maxilla

Figure 2a shows the rectangular region of interest enclosed by landmarks on the anterior cranial base, maxilla and mandible used to detect the keypoints. On the anterior cranial base, this area was defined by the URP, S, Pt and N. On the maxilla, this area was enclosed by the Pt, PNS, ANS and A. On the mandible, this area was enclosed by the LM, Pg, Me and Go.

The ORB’s oriented FAST algorithm [23, 26] was used to detect keypoints on each confined area. We then used the ORB’s steer BRIEF [23] algorithm for keypoint description, a modified algorithm used to solve the problem that BRIEF is not rotationally invariant. Finally, a brute-force Hamming distance [27] was used to match keypoints on two cephalograms.

Considering that the ORB results might not be completely accurate, the Grid-based Motion Statistics for Fast, Ultra-robust Feature Correspondence (GMS) [24] algorithm was applied to filter the matching results. Then, we calculated the relative offset distances and rotation angles of each pair of successfully matched keypoints and transferred the T_2_ cephalogram onto the T_1_ image accordingly. The automated superimposition results were exported as Photoshop files.

Figure 2b shows the flowchart of the automated superimposition process on maxilla.

### Calculation of T2 landmark distances

Photoshop CC 2017 (Adobe, San Jose, CA, USA) was used to register hand tracings onto the corresponding digital cephalograms. The T_1_ cephalograms in each automated superimposition file were used as templates to measure the T_2_ landmark distances (T_2_LDs). The T_1_ tracings were registered onto the templates by 4 positioning reference pinholes. To avoid landmark identification errors, one operator used the automatically superimposed T_2_ cephalograms to identify landmarks by the brush tool with a 3-pixel diameter and noted down the landmarks’ coordinators as automated superimposition results. Then, the T_2_ cephalograms were duplicated, and the corresponding T_2_ tracings were registered on duplicated films at four positioning reference pinholes. Subsequently, they were superimposed onto the templates registered by the bilateral superimposing reference pinholes of the same superimposition type as the automated one. Finally, the landmarks’ coordinators on the duplicated T_2_ cephalograms as the operator’s hand superimposition results were noted down. Before importing the next operator’s tracings, we deleted the tracings of the previous one to prevent mutual influence of the superimposition results among operators.

**Fig. 3:**
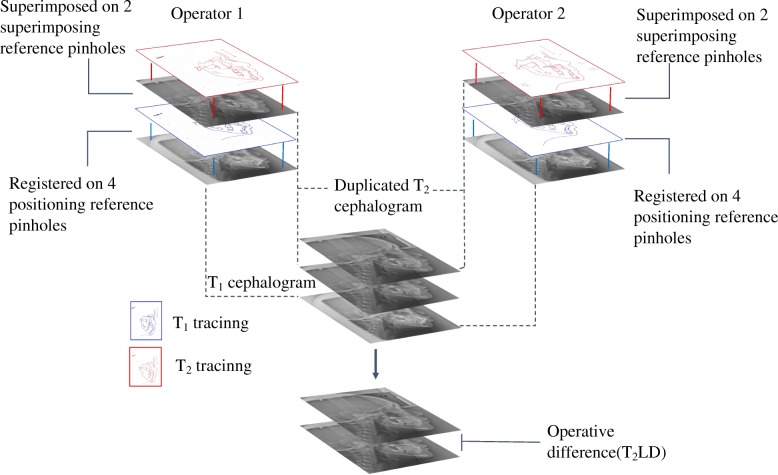
Pairwise T_2_LD for hand superimposition between different operators

To calculate the operative differences of hand superimposition, T_2_LDs between operators’ corresponding coordinators were measured (Fig. 3).

Before evaluating hand superimposition accuracy, the average coordinators among three operators’ coordinators for each landmark were set as the true values. The T_2_LDs between each operator’s coordinators and the corresponding true values were measured.

To evaluate the accuracy of automated superimposition, the T_2_LDs were calculated between the coordinators of automated superimpositions and the corresponding true values.

### Statistical methods

Statistical analyses were carried out with SPSS 25.0 (SPSS Inc., Chicago, IL, USA). The mean T_2_LDs of operative differences in hand superimposition, hand superimposition accuracy and automated superimposition accuracy for multiple cephalometric landmarks under each superimposition method were calculated. A paired t-test was applied to examine the statistical accuracy of automated superimposition with hand superimposition. *P*-values less than 0.05 were considered significant.

## Results

The mean T_2_LDs between operators on the anterior cranial base, maxilla and mandible superimposition methods are listed in Tables 1, 2 and 3, respectively. In the anterior cranial base superimposition (Table 1), the mean T_2_LD at the pterygory point showed the least difference (Pt, 0.61 ± 0.63 mm), while the mean T_2_LD at the menton showed the greatest difference (Me, 1.02 ± 0.96 mm). In the mandibular superimposition (Table 2), the mean T_2_LD at the condylion showed the greatest difference (Co, 1.65 ± 1.24 mm), and the least difference was observed on the LIE (0.62 ± 0.86 mm), followed by the other midline structures (LIA, 0.76 ± 0.55 mm; B point, 0.75 ± 53 mm; Pg, 0.75 ± 0.48 mm; and Me, 0.70 ± 0.45 mm). However, the operator differences in the maxilla were quite close between the landmarks (Table 3), ranging from 0.78 mm to 0.82 mm.

**Table 1:** Mean operative differences in T_2_ landmark distance (mm) between operators. Paired t-test results for the accuracy between hand and automated superimposition, both compared with true values in cranial base superimposition

	N	Operative difference (Hand process) (mm)		N	Accuracy (Hand process) (mm)		Accuracy (Automated process) (mm)		*P*-value
		Mean	SD		Mean	SD	Mean	SD	
Pt	84^a^	0.61	0.63	28	0.33	0.28	0.41	0.37	0.128
PNS	84	0.69	0.65	28	0.40	0.32	0.47	0.39	0.290
ANS	84	0.82	0.76	28	0.47	0.38	0.54	0.45	0.313
A	84	0.85	0.86	28	0.50	0.42	0.59	0.54	0.274
B	84	0.99	0.94	28	0.58	0.46	0.59	0.48	0.880
Me	84	1.02	0.96	28	0.55	0.51	0.64	0.58	0.366
Go	84	0.91	0.88	28	0.52	0.43	0.48	0.50	0.626
Co	84	0.67	0.68	28	0.39	0.34	0.44	0.35	0.254

^a^Because three operators were compared, a total 28 pairs of cephalograms could yield 84 measurement values

**Table 2:** Mean operative differences in T_2_ landmark distance (mm) between operators. Paired t-test results for the accuracy between manual and automated superimposition, both compared with true values in maxillary superimposition

	N	Operative difference (Hand process) (mm)		N	Accuracy (Hand process) (mm)		Accuracy (Automated process) (mm)		*P*-value
		Mean	SD		Mean	SD	Mean	SD	
PNS	84	0.79	0.60	28	0.47	0.25	0.56	0.44	0.189
ANS	84	0.82	0.64	28	0.56	0.37	0.57	0.44	0.855
A	84	0.78	0.56	28	0.54	0.34	0.53	0.45	0.889
UIE	84	0.82	0.63	28	0.51	0.33	0.53	0.38	0.737
UIA	84	0.80	0.58	28	0.49	0.30	0.61	0.61	0.220
UM	84	0.78	0.59	28	0.48	0.27	0.50	0.40	0.718
UMA	84	0.80	0.60	28	0.49	0.35	0.51	0.43	0.763

**Table 3:** Mean operative differences in T_2_ landmark distance (mm) between operators. Paired t-test results for the accuracy between manual and automated superimposition, both compared with true values in mandibular superimposition

	N	Operative difference (Hand process) (mm)		N	Accuracy (Hand process) (mm)		Accuracy (Automated process) (mm)		*P*-value
		Mean	SD		Mean	SD	Mean	SD	
B	84	0.75	0.53	28	0.41	0.25	0.47	0.26	0.254
Pg	84	0.75	0.48	28	0.41	0.22	0.49	0.23	0.059
Me	84	0.70	0.45	28	0.42	0.22	0.46	0.32	0.566
Go	84	1.38	1.21	28	0.74	0.49	0.72	0.39	0.836
Co	84	1.65	1.24	28	0.94	0.59	0.69	0.50	0.695
LIE	84	0.62	0.86	28	0.52	0.36	0.58	0.30	0.538
LIA	84	0.76	0.55	28	0.41	0.25	0.47	0.26	0.226
LM	84	1.00	0.77	28	0.52	0.33	0.58	0.29	0.401
LMA	84	0.87	0.63	28	0.46	0.28	0.52	0.28	0.288

The accuracy of automated superimposition on three superimposition methods by the paired t-test is shown in Tables 1, 2 and 3. Hand superimposition showed slightly higher accuracy compared with paired automated superimposition. However, there were no statistically significant differences (*p* > 0.05) between the two operations in all selected cephalometric landmarks of interest.

## Discussion

Decades ago, researchers studying craniofacial growth in the presence of metallic implants advocated superimposing structures that are stable during growth [18–20], and structural superimposition has been verified as the most accurate technique [21, 22]. However, two remaining issues need to be considered. First, in some films with low quality or high complexity due to overlapping structures, it is impossible for orthodontists to determine a sufficient number of stable structures, especially in the maxilla and mandible. Second, if several stable structures do not fit appropriately in the same between-film position, the orthodontists must defer to their biological knowledge and practical experience. Both of these limitations weaken the reliability of hand structural superimposition.

Landmark identifications are the main source of cephalometric errors [28, 29]. To avoid this type of error and to focus mainly on the reliability and accuracy of superimposition, we carried out our study using film duplication and between-film registration techniques. As shown in Tables 1, 2 and 3, the differences between operators on three types of superimpositions varied greatly from 0.61 mm to 1.65 mm, a range that was greater than the intra-operator reliability reported by Huja et al. [3]. This finding suggests that the inter-operator variability of superimposition is an important variation of results. In our study, we infer that rotational effects produced a greater number of errors than those produced by translational effects on the superimposition of the anterior cranial base and mandible, which were similar to the results from Baumrind et al. [2] and Cook et al. [6]. However, we also found that the mean T_2_LDs of the six landmarks in the maxillary superimposition were quite similar to each other without showing a progressive trend from any centre. This observation suggests that the translational error may have been the predominant cause of error in maxillary superimposition.

The differences in accuracy between hand and automated superimposition are smaller than the variability among operators but also demonstrate a similar increase in error tendency with spatial patterning on the anterior cranial base and mandibular superimpositions. Although slightly higher accuracy errors were observed on the automated superimpositions, there were no significant differences between the two methods in comparison of the differences in accuracy with the true values determined by the paired t-test. This finding suggests that compared with hand tracing, automated superimposition does not lead to a significant increase in error and has the great advantages of absolute consistency and time-efficiency. However, a considerable number of high-quality keypoints would favour better accuracy, which relies on the distinction of stable structures from background by a sharp contrast in pixel intensity. An improvement in image quality, along with advancement of the sensitivity for keypoint detection, would solve the problems of this technique.

From a clinical perspective, considering the time-consuming nature and questionable accuracy of hand cephalometric superimposition when a large number of cases are required for statistical analysis, this automated method could benefit big data analysis using digital cephalograms.

## Conclusions

Computer-aided cephalometric superimposition provides comparable results to those of traditional hand tracing when structural superimposition is concerned. With the help of proper software, this method for digital filmless cephalometry will provide a powerful tool for academic research.

## Data Availability

The full datasets used and analysed during the current study are available on reasonable request from the corresponding authors at tmxuortho@163.com and kqbinghan@bjmu.edu.cn.

## Abbreviations

A: Subspinale
ANS: Anterior nasal spine
B: Supramental
BRIEF: Binary Robust Independent Elementary Features
Co: Condylion
FAST: Features from Accelerated Segment Test
GMS: Grid-based Motion Statistics for Fast, Ultra-robust Feature Correspondence
Go: Gonion
LIA: Lower incisor apex
LIE: Lower incisor edge
LM: Lower first molar mesial buccal cusp
LMA: Lower first molar mesial apex
LRP: Lower reference point
Me: Menton
N: Nasion
ORB: Oriented FAST and Rotated BRIEF
Pg: Pogion
PNS: Posterior nasal spine
Pt: Pterygoid point
S: Sella
T_1_: Pre-treatment
T_2_: Post-treatment
T_2_LDs: T_2_ landmark distances
UIA: Upper incisor apex
UIE: Upper incisor edge
UM: Upper first molar mesial buccal cusp
UMA: Upper first molar mesial apex
URP: Upper reference point
